# Biotransformation and *in Vitro* Metabolic Profile of Bioactive Extracts from a Traditional Miao-Nationality Herbal Medicine, *Polygonum capitatum*

**DOI:** 10.3390/molecules190710291

**Published:** 2014-07-16

**Authors:** Chi-Yu He, Jie Fu, Jing-Yi Ma, Ru Feng, Xiang-Shan Tan, Min Huang, Jia-Wen Shou, Zhen-Xiong Zhao, Xiao-Yang Li, Xian-Feng Zhang, Yangchao Chen, Yan Wang

**Affiliations:** 1 State Key Laboratory of Bioactive Substances and Functions of Natural Medicines, Institute of Materia Medica, Chinese Academy of Medical Sciences, Beijing 100050, China; 2 Department of Neurosurgery, First Hospital, Jilin University, Changchun 130021, China; 3 School of Biomedical Sciences, Faculty of Medicine, The Chinese University of Hong Kong, Shatin, N.T., Hong Kong, China

**Keywords:** *Polygonum capitatum*, extracts, intestinal bacteria, metabolites, LC/MS^n^-IT-TOF

## Abstract

*Polygonum capitatum* Buch.-Ham.ex D. Don, a traditional Miao-nationality herbal medicine, has been widely used in the treatment of various urologic disorders. Recent pharmacological studies demonstrated that a pure compound, FR429, isolated from the ethanol extracts of *P. capitatum* could selectively inhibit the growth of four hepatocellular carcinoma (HCC) cell lines in a dose-dependent manner. Thus, *P. capitatum* probably exhibits potential antitumor activity. However, there is very little information on the metabolism of substances present in *P. capitatum* extracts. In this study, gallic acid, quercetrin, ethanol extracts and ethyl acetate fraction of ethnolic extract (EtOAc fraction) of *P. capitatum* were cultured anaerobically with rat intestinal bacteria. A highly sensitive and selective liquid chromatography electrospray ionization-ion trap-time of fight mass spectrometry (LC/MS^n^-IT-TOF) technique was employed to identify and characterize the resulting metabolites. A total of 22 metabolites (M1–M22), including tannins, phenolic acids and flavonoids, were detected and characterized. The overall results demonstrated that the intestinal bacteria played an important role in the metabolism of *P. capitatum*, and the main metabolic pathways were hydrolysis, reduction and oxidation reactions. Our results provided a basis for the estimation of the metabolic transformation of *P. capitatum in vivo*.

## 1. Introduction

Traditional Chinese medical herbs have been used for medicinal purpose in China for millennia, and they play an important role in Chinese health care. *Polygonum capitatum* Buch.-Ham.ex D. Don, one of the famous traditional Miao-nationality herbal medicine [1], has been shown to possess multiple bioactivities, and it is widely used in the clinic [2,3]. A number of *P. capitatum—*based drugs (e.g., Relinqing Granule and Milin Capsule) have been approved by the China Food and Drug Administration, and are used to treat patients with urologic disorders including pyelonephritis and urinary tract infection [4]. Previous pharmacological studies have demonstrated that ethanol extracts of *P. capitatum* possessed antibacterial, anti-inflammatory, and antioxidant activities [5,6]. Moreover, recent pharmacological studies demonstrated that a typical ellagitannin (ET) FR429, as the most abundant component isolated from ethanol extracts of *P. capitatum* [7], selectively inhibited the growth of four hepatocellular carcinoma (HCC) cell lines, including HepG2, Hep3B, PLC/PRF/5 and Bel-7404, in a dose-dependent manner, whereas its effect on normal liver cells (MIHA) was significantly less [8]. Thus, *P. capitatum* may probably exhibit potential antitumor activity.

In our previous research, besides FR429, we simultaneously determined the other two active constituents in ethanol extracts of *P. capitatum* [7], including gallic acid and quercitrin which possessed a variety of pharmacological activities including sedative, analgesic, antibacterial and anti-invasive effects [9,10]. Although FR429 was studied *in vitro* upon incubation with intestinal bacteria [11], investigations on the comprehensive metabolic profiles of *P. capitatum* extracts have not been reported due to its chemical complexity, the lack of reference compounds, and the inherent limitations of analytical methods.

Studies on the metabolism of *P. capitatum* by rat intestinal bacterial are important for obtaining a better understanding of the biological effects of this herb. In the present study, *P. capitatum* extracts were cultured anaerobically with rat intestinal bacteria, as well as two of the active constituents, gallic acid and quercetrin. HPLC coupled with electrospray ionization (ESI)-ion trap-time of flight mass spectrometry (LC/MS^n^-IT-TOF) was employed to identify and characterize the metabolites. Based on the results, we obtained preliminary knowledge of a possible metabolic pathway for these extracts in intestinal flora *in vitro*, and then we discussed the differences between multi-component extracts and the single component in the intestinal flora metabolic transformation in order to provide a foundation for further exploring the efficacy and toxicity of *P. capitatum*.

## 2. Results and Discussion

### 2.1. Identification of the Metabolites of Gallic Acid in Rat Intestinal Bacteria

**Figure 1:**
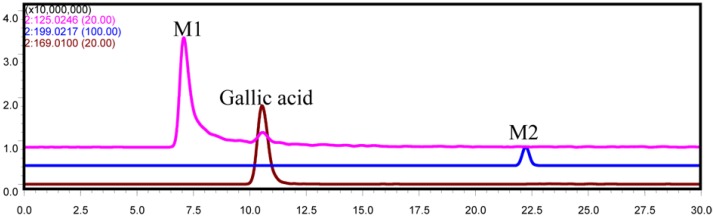
Extracted ion chromatograms (EICs) of metabolites in the incubation of gallic acid in the intestinal bacteria *in vitro* (negative ion mode, the *m/z* of gallic acid and its metabolites in this chromatogram: gallic acid, 169.0136; M1, 125.0246; M2, 199.0217).

Two metabolites together with the parent drug were detected by comparison with the blank sample (Figure 1). The metabolites were marked as M1 and M2 according to their retention times. They were detected both in positive negative ion mode and showed good responses (Figure 1).

**Table 1:** LC/MS^n^ data obtained for gallic acid and its metabolites from intestinal flora incubation *in vitro* (positive and negative ion mode).

	t_R_ (min)	Positive Mode	Negative Mode		
		MS^1^ [M + H]^+^	MS^1^ [M − H]^−^	MS^2^	MS^3^
Gallic acid	10.5	171.0265	169.0136	125.0258	
M1	7.2	127.0321	125.0246	81.0383, 97.0355, 107.0201	--
M2	22.0	201.1478	199.0217	153.0217	109.0328

The parent drug (t_R_ = 10.5 min) had a [M + H]^+^ at *m/z* 171.0265 and a [M − H] ^−^ at *m/z* 169.0140, and the mass spectral data of the parent drug and metabolites were listed in Table 1.

M1 (t_R_ = 7.2 min) had a [M + H]^+^ at *m/z* 127.0321 and a [M − H]^−^ at *m/z* 125.0246. Regarding the molecular ion in positive mode ([M + H]^+^), the *m/z* value of M1 was 44 Da less than that of the parent drug. These results demonstrated that M1 was formed via a loss of a CO_2_ group from gallic acid. Therefore, M1 was inferred to be 1,2,3-trihydroxybenzene, also known as pyrogallic acid.

M2 (t_R_ = 22.0 min) had a [M + H]^+^ at *m/z* 201.1478 and a [M − H]^−^ at *m/z* 199.0217. The *m/z* value of M2 was 30 Da more than that of the parent drug, suggesting that M2 could be a methoxyl compound derived from gallic acid via an oxidation reaction. Thus, M2 was inferred to be a methoxy-derivative of gallic acid.

**Scheme 1:**
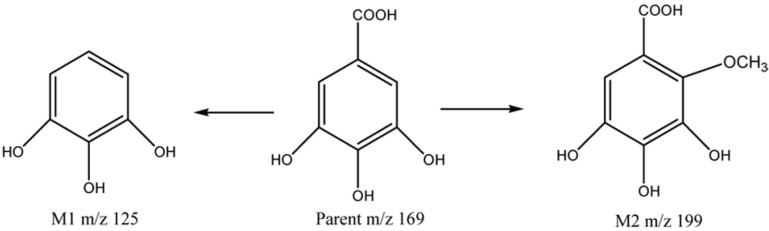
Metabolic pathway of gallic acid in intestinal flora incubation *in vitro.*

In summary, two metabolites of gallic acid were identified in the rat intestinal bacteria incubation system, namely pyrogallic acid (M1) and a gallic acid methoxyl compound (M2). The proposed metabolic pathway of gallic acid was shown in Scheme 1.

### 2.2. Identification of the Metabolites of Quercetrin in Rat Intestinal Bacteria

**Figure 2:**
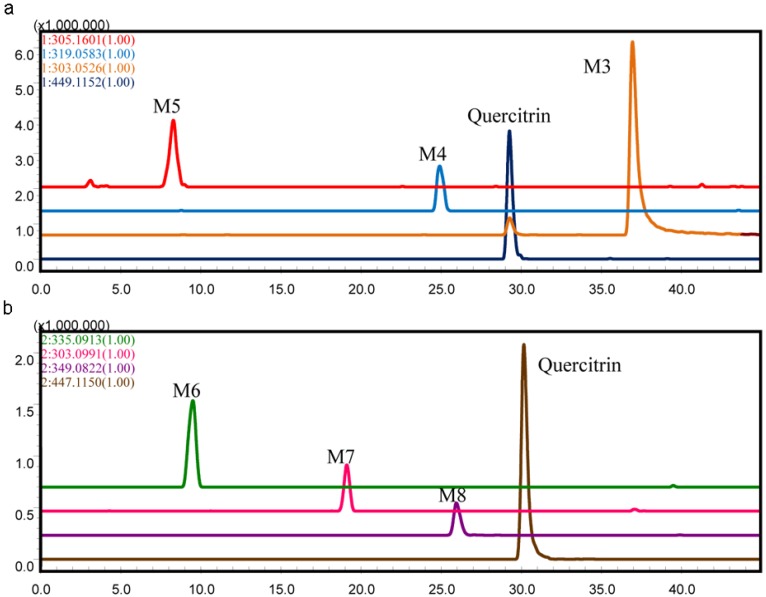
Extracted ion chromatograms (EICs) of metabolites in the incubation of quercitrin in the intestinal bacteria *in vitro* (**a**) positive ion mode, the *m/z* of quercitrin and its metabolites in this chromatogram: quercitrin, 449.1152; M3, 303.0526; M4, 319.0583; M5, 305.1601; (**b**) negative ion mode, the *m/z* of quercitrin and its metabolites in this chromatogram: quercitrin, 447.1150; M6, 335.0913; M7, 303.0991, M8, 349.0822).

Six metabolites together with the parent drug were detected by comparison with the blank sample (Figure 2). The metabolites were marked as M3–M8 according to their HPLC retention times and extracted ion chromatograms (EICs). Both positive and negative ESI were used. M3, M4, and M5 showed good responses in positive ion mode, whereas the others were detected in negative ion mode (Figure 2b).

The parent drug (t_R_ = 29.3 min) had a [M + H]^+^ at *m/z* 449.1082 and a [M − H]^−^ at *m/z* 447.0908, which was consistent with previously published results [12].

M3 (t_R_ = 36.4 min) had a [M + H]^+^ at *m/z* 303.0501, and the MS^2^ spectrum had fragments at *m/z* 257.0431 (M − 46 Da, loss of a H_2_O and a CO), *m/z* 229.0471 (M − 46 Da − 28 Da, loss of a H_2_O and 2 CO), and *m/z* 201.0520 (M − 46 Da − 28 Da − 28 Da, loss of a H_2_O and 3 CO). When the *m/z* 303.0501 ion further fragmented in the MS^3^ spectrum, it produced an ion at *m/z* 173.0621 (M − 46 Da − 28 Da − 28 Da − 28 Da, loss of a H_2_O and 4 CO). Compared to the fragmentations of the parent drug (M − 146 Da − 46 Da − 28 Da − 28 Da − 28 Da, loss of a sugar moiety, a H_2_O and 4 CO), M3 might be formed through a loss of a sugar moiety. On the other hand, as previously reported, quercetin had a positively charged molecular ion at *m/z* 303 and yields MS^n^ at *m/z* 257, 299, 201, and 173 [12]. Thus, M3 was identified as quercetin, which was the key metabolite of quercetrin.

**Scheme 2:**
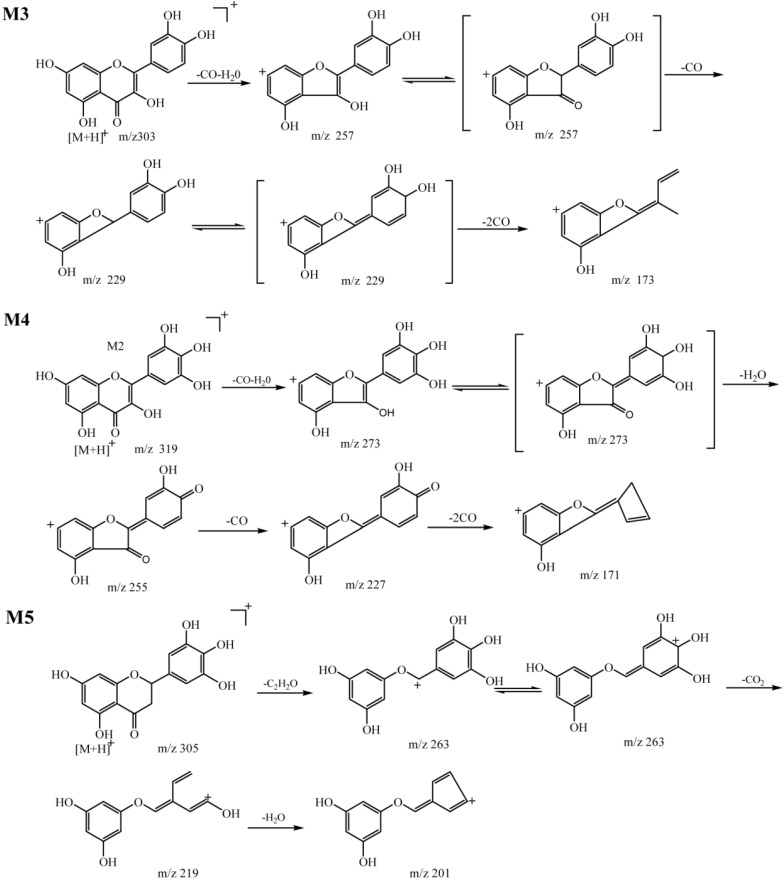
Fragmentation pathway of metabolites M3, M4 and M5 (positive ion mode).

M4 (t_R_ = 25.2 min) had a [M + H]^+^ at *m/z* 319.0431, and the MS^2^ spectrum had fragments at *m/z* 273.0386 (M − 46 Da, loss of a H_2_O and a CO). MS^3^ of *m/z* 319.0431 produced major ions at *m/z* 255.0227 (M − 46 Da − 18 Da, loss of 2 H_2_O and a CO) and *m/z* 227.0324 (M − 46 Da − 18 Da − 28 Da, loss of 2 H_2_O and 2 CO). MS^4^ of *m/z* 319.0431 produced a major ion at *m/z* 171.0440 (M − 46 Da − 18 Da-28 Da − 46Da, loss of 3 H_2_O and 3 CO). Comparing the fragmentation data for M4 and M3, we observed two important points: one was that, the *m/z* value of M4 was 16 Da more than that of M3, suggesting that M4 obtained a hydroxyl group via an oxidation reaction; the other one was that the fragmentations of M4 and M3 were very similar to each other. For example, a comparison of the fragments at *m/z* 257.0431, 229.0471, and 173.0621 of M4 with those at *m/z* 255.0227, 227.0324, and 171.0440 of M3, which was only 2 Da different, demonstrated that the fragmentation pathways of the two metabolites were similar (Scheme 2). Therefore, these results indicated that ring B was substituted at the C-5 position with a hydroxyl group and M4 was inferred to be 3,5,7,3',4',5'-hexahydroxyflavone, which was previously isolated from *Thuja occidentalis* [13]. 

M5 (t_R_ = 8.6 min) had a [M + H]^+^ at *m/z* 305.0105, and the MS^2^ spectrum had fragments at *m/z* 263.9851 (M − 42 Da, loss of a C_2_H_2_O). The MS^3^ spectrum had fragments at *m/z* 219.9994 (M − 42 Da − 44 Da, loss of a C_2_H_2_O and a CO_2_), whereas MS^4^ yielded a major ion at *m/z* 201.9893 (M − 42 Da − 44 Da − 18 Da, loss of a C_2_H_2_O, a CO_2_, and a H_2_O). Based on the fragmentation data, the *m/z* value of M5 was 2 Da more than that of M3, thus, M5 might be transformed by M3 via reduction reactions. According to the characteristic fragmentation behaviors mentioned above, M5 was 5,7,3',4',5'-pentahydroxylflavanone, which was isolated from *Euryale Ferox* seed [14]. The fragmentation pathways were shown in Scheme 2.

M6 (t_R_ = 8.9 min) had a [2M − H]− at *m/z* 355.0576, and MS2 yielded a molecular ion ([M − H]−) at *m/z* 167.0202 (M − 168 Da), suggesting that M4 was generated by an oxidation reaction of quercetrin in ring-B after retro-Diels-Alder (RDA) cleavage. Hence, M6 was inferred to be 3,4-dihydroxy-benzeneacetic acid, which has been previously reported as a metabolite of quercetrin [15]. M7 (t_R_ = 18.5 min) had a [2M − H]− at *m/z* 303.0997, and MS2 yielded a molecular ion ([M − H]−) at *m/z* 151.0409 (M − 152 Da). The *m/z* value of M7 was 16 Da less than that of M6, and M7 was identified as 3-hydroxybenzeneacetic acid, which was obtained via a reduction reaction of M6 and reported as a metabolite of quercetrin [15]. M8 (t_R_ = 25.2 min) had a [M − H]^−^ at *m/z* 349.0677, and the MS^2^ spectrum had fragments at *m/z* 331.0624 (M − 18Da, loss of a H_2_O) and *m/z* 299.0349 (M − 18 Da − 32 Da, loss of a H_2_O and a O_2_). The MS^3^ spectrum had fragments at *m/z* 271.0370 (M − 18 Da − 32 Da − 28 Da, loss of a H_2_O, a O_2_, and a CO), and MS^4^ of *m/z* 349.0677 produced a major ion at *m/z* 227.0469 (M − 18 Da − 32 Da − 28 Da − 44 Da, loss of a H_2_O, a O_2_, a CO, and a CO_2_). The *m/z* value of M8 was 48 Da more than that of M3, and in this case, M8 could be a multi-hydroxylation compound derived from M1 via hydroxylation reaction with three more hydroxyl groups. 

**Scheme 3:**
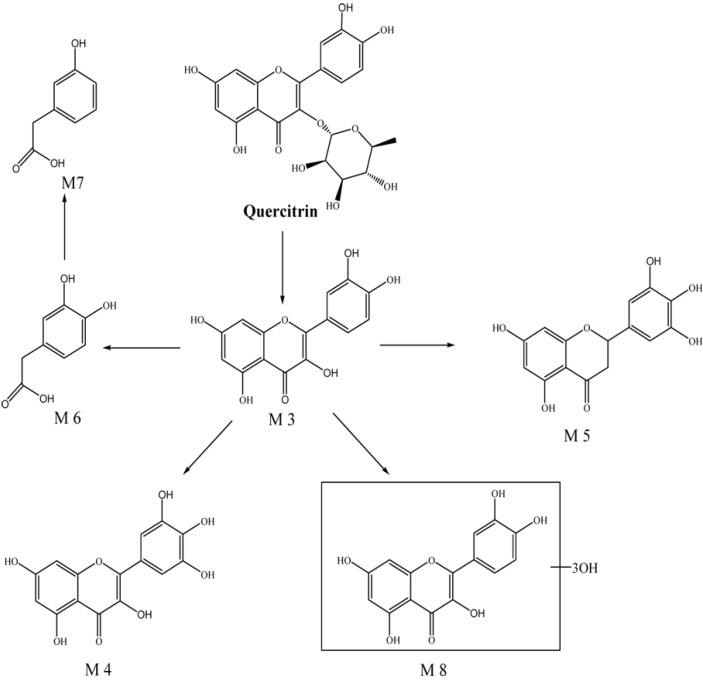
Metabolic pathway of quercitrin in intestinal flora incubation *in vitro.*

In summary, six metabolites of quercetrin were isolated from rat intestinal bacteria cultures. Three of them were identified as previously reported metabolites of quercetrin [e.g., quercetin (M3), 3,4-dihydroxybenzeneacetic acid (M6) and 3-hydroxybenzeneacetic acid (M7)]. The other three metabolites [e.g., 3,5,7,3',4',5'-hexahydroxyflavone (M4), 5,7,3',4',5'-pentahydroxylflavanone (M5), and multi-hydroxylation of quercetin (M8)] were known as flavonoid compounds but were reported as metabolites of quercetrin for the first time in this study. The identifications of M3–M8 together with the parent drug, based on fragmentation data, were summarized below and presented in Table 2 and . On the basis of the identification of these metabolites, the proposed metabolic pathway of quercetrin was inferred (Scheme 3). 

**Table 2:** LC/MS^n^ data obtained for quercitrin and its metabolites from intestinal flora incubation *in vitro* (positive ion mode).

	t_R_ (min)	MS^1^ [M + H]^+^	Fragments		
			MS^2^ *m/z*	MS^3^ *m/z*	MS^4^ *m/z*
**Parent**	29.3	449.1152	303.0466	229.0449, 201.0470 257.0414, 201.0470 165.0127, 137.0179	229.0433,161.0665
**M3**	36.4	303.0526	229.0471, 257.0495 201.0520, 285.0419 247.0630, 165.0204	229.0515, 201.0571 173.0621, 187.0422 161.0633, 155.0528 145.0680, 183.0498	145.0738
**M4**	25.2	319.0583	273.0386, 247.0575 165.0171, 357.0452	255.0227, 227.0324 245,0383, 177.0471 199.0415, 217.0444 171.0440	171.0492,227.0270
**M5**	8.6	305.1601	263.9851	219.9817, 201.9733	201.9720

**Table 3:** LC/MS^n^ data obtained for quercitrin and its metabolites from intestinal flora incubation *in vitro* (positive ion mode).

	t_R_ (min)	MS^1^ [2M − H]^−^	MS^1^ [M − H]^−^	Fragments		
				MS^2^ *m/z*	MS^3^ *m/z*	MS^4^ *m/z*
**Quercitrin**	29.3	--	447.1150	301.0382, 297.0411, 273.0605, 179.0104, 151.0135	273.0311, 255.0285, 229.0608, 283.0361, 179.0084, 271.0364	227.0424, 243.0424, 215.0357, 199.0460, 171.0512
**M6**	8.9	335.0913	167.0403	167.0403, 123.0524	--	--
**M7**	18.5	303.0991	151.0409	151.0409 107.0409	--	--
**M8**	25.2	--	349.0822	331.0624 299.0349 271.0436 179.0073, 151.0088, 191.0122	299.0321, 271.0370, 179.0137,227.0469	271.0324, 255.0408, 227.0469

Our findings regarding on quercetrin metabolism were agreed with those in a previous study reporting that quercetin (M3), 3,4-dihydroxybenzeneaqcetic acid (M6), and 3-hydroxybenzeneacetic acid (M7) were metabolites of quercetrin [15], even though the methods used in the two studies were different. TLC, EI-MS and human intestinal bacteria were applied in their study, whereas LC/MS^n^-IT-TOF and rat intestinal bacteria were employed in our study.

### 2.3. Identification of the Metabolites of Ethanol Extracts and EtOAc Extracts Fraction of P. capitatum in Rat Intestinal Bacteria

**Figure 3:**
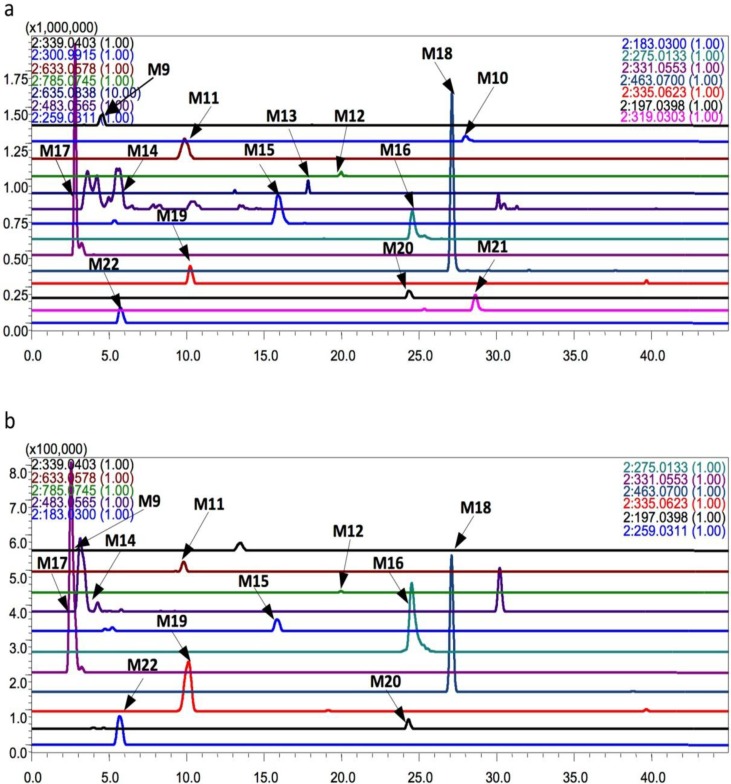
Extracted ion chromatograms (EICs) of metabolites in the incubation of extracts of *P. capitatum* in the intestinal bacteria *in vitro* (**a**) EICs of metabolites of EtOAc fraction (negative ion mode); (**b**) EICs of metabolites of ethanol extracts (negative ion mode), the *m/z* of metabolites in these two chromatograms: M9, 339.0281; M10, 300.9915; M11, 633.0578; M12, 785.0745; M13, 635.0838; M14, 483.0565; M15, 183.0300; M16, 275.0133; M17, 331.0553; M18, 463.0700; M19, 335.0623; M20, 197.0398; M21, 319.0303; M22, 259.0311.

Through comparisons with the control group cultured at 0 h (Figure 3), a total of 18 metabolites of EtOAc fraction were detected in rat intestinal bacteria by LC/MS^n^-IT-TOF. The metabolites were marked as M9–M22 according to their retention times. Thirteen of them were metabolites of FR429, namely M9 (t_R_ = 4.5 min), M10 (t_R_ = 27.7 min), M11(t_R_ = 9.8 min), M12 (t_R_ = 19.9 min), M13 (t_R_ = 17.8 min), M14-1 (t_R_ = 3.6 min), M14-2 (t_R_ = 4.2 min), M14-3 (t_R_ = 5.4 min), M14-4 (t_R_ = 7.7 min), M14-5 (t_R_ = 8.2 min), M14-6 (t_R_ = 8.2 min), M15 (t_R_ = 16.5 min), M16 (t_R_ = 24.5 min), and M17 (t_R_ = 3.1 min). The others were metabolites of quercetrin, namely M18 (t_R_ = 26.4 min), M19 (t_R_ = 9.5 min), M20 (t_R_ = 23.7 min), M21 (t_R_ = 28.4min), and M22 (t_R_ = 5.7 min). Meanwhile, 11 metabolites of the ethanol extracts were detected. Seven of them were metabolites of FR429, namely M9, M10, M11, M12, M13, M14-1, M14-2, M15, M16, and M17, whereas four of them were metabolites of quercetrin, namely M18, M20, M21, and M22. They were detected in negative ion mode and showed good responses. The EIC results were shown in Figure 3, and the mass fragmentation data were summarized in Table 4.

In our previous research, we studied the metabolism of FR429 by intestinal bacteria *in vitro* and identified 13 metabolites [11]. According to comparisons of their accurate molecular masses and fragment ions at each MS^n^ stage with those of the extracts in the current study, M9–M16 were identified and the data were shown in Table 4 In addition, M17 had a [M + H]^+^ at *m/z* 331.0553, and the MS^2^ spectrum had fragments at *m/z* 313.0049 (M − 18 Da, loss of a H_2_O) and *m/z* 169.0114 (M − 162 Da, loss of a glucose). Compared with the fragmentation of the molecular ion ([M + H]^+^) of M14 that yielded MS^2^ at *m/z* 313.0449 through loss of the sugar moiety, M17 was obtained by the loss of four galloyl groups from FR429. Another five were metabolites of quercetrin. M18 had a [M − H]^−^ at *m/z* 463.0700, and the *m/z* value of M18 was 16 Da more than that of quercetrin, suggesting that M18 was formed by addition of a hydroxyl group from quercetrin via oxidation reaction.

**Table 4:** LC/MS^n^ data obtained for extracts of *P. capitatum* and its metabolites from intestinal flora incubation *in vitro* (negative ion mode).

	t_R_ (min)	MS^1^ [M − H]^−^	Fragments		Name	Obtained from	Metabolite from
			MS^2^	MS^3^			
**FR429**	25.4	468.0410(2)	446.0485(2), 275.0201, 300.9967, 169.0164, 370.0372(2)	--		botanical extracts	
**Quercitrin**	30.3	447.0764	301.0220	150.9999,178.9931, 255.0174,271.0134		botanical extracts	
**M9**	4.5	339.0403	169.0125	125.0258	gallic acid	botanical extracts and intestinal flora incubation	EtOAc fraction and ethanol extract
**M10**	27.7	300.9915	229.0151, 257.0123	--	ellagic aicd	intestinal flora incubation	EtOAc fraction
**M11**	9.8	633.0578	300.9911	229.0085,257.0019	helioscopinin B	intestinal flora incubation	EtOAc fraction and ethanol extract
**M12**	19.9	785.0745	465.0487, 571.0460	--	d-glucopyranose	intestinal flora incubation	EtOAc fraction and ethanol extract
**M13**	17.8	635.0838	483.0635	193.0140	three galloyls glucose	intestinal flora incubation	EtOAc fraction
**M14-1**	3.6	483.0686	331.0575, 169.0117, 313.0478	--	two galloyls glucose	intestinal flora incubation	EtOAc fraction and ethanol extract
**M14-2**	4.2	483.0653	331.0555, 313.0486, 169.0151	--	two galloyls glucose	intestinal flora incubation	EtOAc fraction and ethanol extract
**M14-3**	5.4	483.0666	331.0599, 313.0457, 169.0129	--	two galloyls glucose	intestinal flora incubation	EtOAc fraction
**M14-4**	7.7	483.0784	313.0449, 183.0363, 169.0074	--	two galloyls glucose	intestinal flora incubation	EtOAc fraction
**M14-5**	8.2	483.0784	313.0449, 183.0363, 169.0074	--	two galloyls glucose	intestinal flora incubation	EtOAc fraction
**M14-6**	10.4	483.0585	423.0437, 271.0383, 169.0074	--	two galloyls glucose	intestinal flora incubation	EtOAc fraction
**M15**	16.5	183.0300	169.0124	--	methyl-derivative of gallic acid	intestinal flora incubation	EtOAc fraction and ethanol extract
**M16**	24.5	275.0133	257.0021	229.0089, 201.0134	3,4,8,9,10-pentahydroxydibenzo-[b,d]pyran-6-one	intestinal flora incubation	EtOAc fraction and ethanol extract
**M17**	3.1	331.0553	169.0114, 313.0049	--	galloyl glucose	intestinal flora incubation	EtOAc fraction
**M18**	26.4	463.0700	317.0114	271.0138, 287.0083, 178.9943, 151.0013	myricetrin	intestinal flora incubation	EtOAc fractionand ethanol extract
**M19**	9.5	335.0623	167.0275	123.0420	3,4-dihydroxybenzeneacetic acid	intestinal flora incubation	EtOAc fraction and ethanol extract
**M20**	23.7	197.0398	169.0093	125.0211	4,6-dihydroxy-2-ethoxy benzoic acid	intestinal flora incubation	EtOAc fractionand ethanol extract
**M21**	28.4	319.0303	272.9924	244.9958, 217.0104 201.0243	1-(3,4-dihydroxyphenyl)-2-hydroxy-3-(2,4,6-trihydroxy-phenyl)-1,3-propanedione	intestinal flora incubation	EtOAc fraction
**M22**	5.7	259.0311	151.0157	--	1-(2',4',6'-hydroxy)-3-phenyl-propan-1-one	intestinal flora incubation	EtOAc fraction and ethanol extract

The MS^2^ spectrum had fragments at *m/z* 317.0114 (M – 146 Da, loss of a rhamnose). Compared to the fragmentation data for M18 from quercetrin, the MS^3^ spectrum had the same fragments at *m/z* 271.0138 (M − 146 Da − 46 Da, loss of a rhamnose, a H_2_O, and a CO), 178.9943 (M − 146 Da − 46 Da − 92 Da), and 151.0013 (M − 146 Da − 46 Da − 92 Da − 28 Da). In addition, the fragment at *m/z* 287.0083 may have formed from a hydroxyl group (16 Da) obtained from the fragment at *m/z* 271.0138. Based on the fragmentation data and previous reports, M18 was identified as a hydroxylated form of quercetrin known as myricetrin. 

M19 had a [2M − H]^−^ at m/z 355.0623, and MS2 yielded a molecular ion ([M − H]^−^) at m/z 167.0275 (M − 168 Da), suggesting that M19 was generated via an oxidation reaction of quercetrin in ring-B after RDA cleavage. Therefore, M19 was identified as 3,4-dihydroxybenzeneacetic acid, which also was detected as a metabolite of quercetrin in rat intestinal bacteria. M20 had a [M − H]^−^ at m/z 197.0303, and the MS^2^ spectrum had fragments at m/z 169.0093 (M − 28 Da, loss of a CO). These results indicated that M20 may be a derivative of gallic acid. According to the fragmentation data, M20 was inferred to be 4,6-dihydroxy-2-ethoxybenzoic acid. 

M21 had a [M − H]^−^ at *m/z* 319.0303, and the MS^2^ spectrum had fragments at *m/z* 272.9924. MS^3^ of *m/z* 319.0303 produced major ions at *m/z* 244.9958 and *m/z* 201.0243 Comparisons of the fragments of M21 with those of quercetin, which had fragments at *m/z* 301, 255, 227, and 183 revealed that there was an 18-Da difference between M21 and quercetin. Thus, M21 was formed from quercetin via a hydrolysis reaction and identified as 1-(3,4-dihydroxyphenyl)-2-hydroxy-3-(2,4,6-trihydroxyphenyl)-1,3-propanedione. M22 had a [M − H]^−^ at *m/z* 259.0311, and MS^2^ yielded a major ion at *m/z* 151.0157, which was the characteristic ion of flavonoids after RDA cleavage. Thus, M22 was inferred to be 1-(2',4',6'-hydroxy)-3-phenylpropan-1-one.

**Scheme 4:**
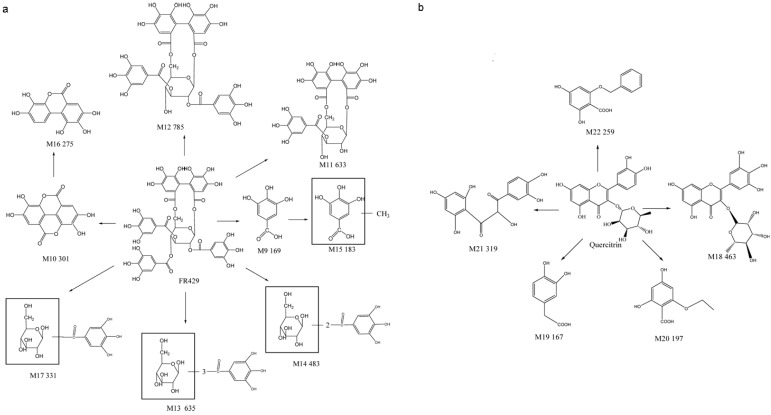
Metabolic pathways of extracts of *P. capitatum* in intestinal flora incubation *in vitro.* (**a**): metabolite pathway of FR429 in extracts; (**b**): metabolite pathway of quercetrin in extracts.

The metabolites of FR429 within extracts by rat intestinal bacteria were similar to those of FR429, but fewer metabolites were obtained. Further dehydroxylation could be observed in the metabolism of pure compound, but not in the extracts. According to the structures of the metabolites, the metabolites of FR429 within extracts were mainly generated by hydrolysis and reduction reactions in intestinal flora, and the galloyl groups were easily removed from C-2 and/or C-4 of glucose. Whereas, the metabolites of quercetrin in extracts were quite different with those of pure compound. Only 3,4-dihydroxy-benzeneacetic acid (M6 and M19) was detected both in the metabolites of quercetrin in extracts and those of pure quercetrin. 3,5,7,3',4',5'-hexahydroxyflavone (M4) and myricetrin (M18) have similar structures, except that the ring C was substituted at the C-3 position with a rhamnose group of M18. However, the structures of M5 and M8 were different from those of M20, M21, and M22. The formers were hydroxyl derivatives of quercetrin obtained via oxidation reaction, whereas the latters were generated by cleavage of a sugar moiety in ring C. The proposed metabolic pathway of the extracts in rats intestinal bacteria were shown in Scheme 4. As described above, we speculated that the qualitative difference of the metabolites between *P. capitatum* extract and pure compound(s) was possibly due to that several kinds of compounds were metabolized by similar metabolism enzymes, but with different affinities to the enzymes. For example, the methylated metabolite of EA, but not GA, can be found in the *in vitro* incubation of FR429 with primary rat hepatocytes in our previous study, which was probably caused by the low affinity of GA to the metabolism enzyme COMT [16] Therefore, extensive studies of *P. capitatum* on the intestinal bacterial communities are necessary. Extensive metabolism by the intestinal microflora provided metabolites that were better absorbed with potential bioactivity. The bioavailability of ellagitannins was very low, whereas theirs metabolites, with smaller molecular weights, like ellagic acid and urolithins, had better absorption [17]. Ellagic acid and its metabolites, including dimethyl EA and urolithins, have been shown to exhibit potential anti-inflammatory, anti-cancer, antimicrobial, and antioxidant activities *in vitro* and *in vivo* [18,19]. Quercetin was the major metabolite of quercitrin, and it was reported that the absorption rate and bioavailability of aglycone quercetin were higher than that of quercetin glycosides [20,21]. Thus, the production of quercetin could improve the absorption rate and bioavailability of quercitrin *in vivo*. 3, 4-Dihydroxybenzeneacetic acid, observed both in the metabolites of extracts and pure compound, has shown antioxidant, antimicrobial, antiviral, and capability for treating central nervous system diseases treatment activity [22]. Therefore, the metabolites of the extracts by intestinal flora may have their potential contribution to the pharmacologic activity of *P. capitatum*.

## 3. Experimental Section

### 3.1. Materials and Reagents

*P. capitatum* was collected from Guiyang City, Guizhou Province and identified by Professor Ma Lin of the Institute of Materia Medica, Chinese Academy of Medical Sciences. Quercetrin (No. 111538-200504, purity > 98%) and gallic acid (No. 110831-200302, purity > 98%) were purchased from the National Institute for the Control of Pharmaceutical and Biological Products (Beijing, China). Beef extract, peptone, and nutrient agar were supplied by Beijing Aoboxing Biotech Company Ltd. (Beijing, China) HPLC grade acetonitrile, methanol, and the other analytical grade chemical reagents were purchased from Beijing Chemical Reagent Co., Ltd. (Beijing, China). Distilled water was Wahaha purified water.

### 3.2. Apparatus

An HPLC-ESI-IT-TOF mass spectrometer (Shimadzu, Kyoto, Japan) equipped with a DGU-20A_3_ degasser, a LC-20AD binary pump, a SIL-20AC auto sampler, a CTO-20A column oven, a SPD-M20A diode array detector. A liquid chromatography mass spectrometer (LCMS) with LCMS solution software was used to detect and identify the parent drug and its metabolites via on-line analysis.

### 3.3. Animals

Sprague-Dawley rats (male, 200–250 g) were obtained from the Experimental Animal Science Department of Peking University Health Science Center (Beijing, China) with the license No. SCXK (Beijing, China) 2006-0008 and housed with free access to a normal standard chow diet and water. All animals were maintained on a 12 h light/dark cycle (lights on from 8:00 AM to 8:00 PM) at ambient temperature (20–25 °C) with 40%–70% relative humidity. Animals were provided with sawdust bedding material and were housed under these conditions for at least 1week prior to the experiments. Rats were fasted for 12 h before all experimental studies.

### 3.4. Preparation of the Anaerobic Medium Broth and Intestinal Bacteria Culture Solution

The anaerobic medium broth for intestinal bacteria culture was prepared according to a previously published method [23]. Briefly, the following solutions were prepared: solution A (0.78% K_2_HPO_4_), solution B (0.47% KH_2_PO_4_, 1.18% NaCl, 1.2% (NH_4_)_2_SO_4_, 0.12% CaCl_2_, and 0.25% MgSO_4_·H_2_O), solution C (8% Na_2_CO_3_), solution D (0.5 g L-cysteine, 1 g beef extract, 1 g peptone, and 1 g nutrient agar dissolved in 20 mL distilled water), and a solution of 25% L-ascorbic acid. Then a mixture was prepared from 37.5 mL solution A, 37.5 mL solution B, 50 mL solution C, 20 mL solution D, and 2 mL L-ascorbic acid solution. After the pH of the mixture was adjusted to approximately 7.5, the total solution was transferred to a 1-L volumetric flask, and the final volume adjusted to 1000 mL with distilled water. The obtained anaerobic medium was autoclaved at 0.1 MPa and 121 °C for 20 min in tubes, and then it could be used immediately after cool-down or stored at 4 °C for 1 month. 

Intestinal bacteria culture solution was prepared according to a previously published method [18]. After sacrificed by cervical dislocation under anesthesia, rat’s abdomon was incised, and colonic material was immediately extracted in an anaerobic chamber. The fresh feces were homogenized under anaerobic condition, and 1 g of them was transferred to a flask containing 20 mL anaerobic medium, which was degassed with N_2_ sparging. After mixing, cultures were incubated under anaerobic conditions of a N_2_ atmosphere at 37 °C for 60 min, and then the culture solution containing intestinal bacteria was collected. 

### 3.5. Sample Preparation

Preparation of ethanol extracts of *P. capitatum*/Ethanol extracts were prepared by macerating 1 g of *P. capitatum* powder with 50 mL of 80% ethanol for 12 h at room temperature, followed by ultrasonic extraction for 30 min. After filtration, the residue was washed with ethanol, and the combined filtrate was concentrated by rotary evaporation and dried at room temperature. 

Preparation of ethyl acetate fraction of ethnolic extract (EtOAc fraction) of *P. capitatum*/EtOAc fraction were prepared by macerating 1 g of *P. capitatum* powder with 50 mL of 80% ethanol for 12 h at room temperature, followed by ultrasonic extraction for 30 min. After filtration, the residue was washed with ethanol, and the combined filtrate was concentrated by rotary evaporation. Then, the concentrated filtrate was extracted four times with an equal volume ethyl acetate, and the combined filtrate was dried. 

Metabolism of gallic acid, quercetrin, and extracts *in vitro* and sample preparation: gallic acid (10 mg) and quercetrin (10 mg) were weighed and dissolved in 100 μL methanol. Ethanol extracts and EtOAc fraction were dissolved in 200 μL methanol, respectively. Solutions of gallic acid (10 μL), quercetrin (10 μL), ethanol extracts (10 μL), and EtOAc fraction (10 μL) were added to individual samples of fresh intestinal bacteria solution (1 mL), and methanol (10 μL) was added as a negative control. The cultures were incubated at 37 °C for 0, 6, 12, 18, 24, 36, 48, and 72 h. After the reactions were terminated with 2% formic acid (100 μL) and acetonitrile (1 mL), samples were mixed using a vortex mixer for 30 s and then centrifuged at 10,000 ×*g* for 15 min. The supernatant was dried under N_2_ flow at room temperature, and then the residue was dissolved in 500 μL methanol and filtered through a 0.22-μm micropore membrane. Ten-microliter samples of each culture solution were analyzed by LCMS-IT-TOF.

### 3.6. Analytical Conditions

A high performance liquid chromatography coupled to an IT-TOF mass spectrometer (Shimadzu, Kyoto, Japan) was used in this study. An Alltima C_18_ column (150 × 4.6 mm, 5 μm) was used, and the mobile phase consisted of eluent A (0.2% formic acid in water, v/v) and B (acetonitrile). A gradient elution was employed for the separation. The mobile phase for gallic acid was programmed as follows: an isocratic elution of 5% eluent B for the first 10 min, followed by a linear gradient elution of 5%–10% eluent B from 10 to 20 min, and 10%–30% eluent B from 20 to 30 min. The flow rate was 0.8 mL·min^−1^. The detection wavelength range and the column temperature were set at 215 nm and 30 °C, respectively. The mobile phase for quercetrin and extracts was programmed as follows: an isocratic elution of 10% eluent B for the first 10 min, followed by a linear gradient elution of 10%–30% eluent B from 10 to 30 min, 30%–55% eluent B from 30 to 40 min, and 55%–70% eluent B from 40 to 45 min. The flow rate was 0.8 mL·min^−1^. The detection wavelength range and the column temperature were set at 254 nm and 30 °C, respectively. 

For HPLC-MS analysis, an IT-TOF mass spectrometer (Shimadzu) was connected to the Shimadzu UFLC system via an ESI interface. Both positive and negative ESI were used to analyze the metabolites, and the parameters were as follows: CDL temperature, 200 °C; heat block temperature, 200 °C; detector voltage, 1.75 kV; nebulizing gas flow rate, 1.5 L/min; and drying gas pressure, 120 kPa. The ion accumulation time and precursor ion isolation width were set at 20 ms and 3 Da, respectively. Mass spectra were acquired in the range of *m/z* 100–800 for MS^1^. The MS^n^ data were collected in an automatic mode. The collision-induced dissociation (CID) energy was set at 50%.

## 4. Conclusions

LC/MS^n^-IT-TOF was applied for on-line *in vitro* analysis of the metabolites of gallic acid, quercitrin, and extracts of *P. capitatum* in intestinal flora in our study. A total of 22 metabolites were detected and characterized. Our results illustrated the value of LC/MS^n^-IT-TOF as a highly sensitive and accurate tool for analyzing trace metabolites in complex biological systems depending on multi-stage mass information. It was clear that LC/MS^n^-IT-TOF was an excellent tool for the identification of natural products. Furthermore, we studied the biotransformation and metabolic profiles of extracts of *P. capitatum* in cultured intestinal flora *in vitro* for the first time. The overall results demonstrated that *P. capitatum* could be easily transformed by intestinal bacteria *in vitro*, and the main metabolic pathways involved hydrolysis, reduction and oxidative reactions. We speculated that the qualitative difference of the metabolites between *P. capitatum* extract and pure compound(s) was possibly due tothe metabolic enzymes. Therefore, further research will be performed on the enzyme system which may contribute to the bacterial metabolism of *P. capitatum*. In conclusion, the intestinal bacteria played an important role in the metabolism of *P. capitatum*, and our results provided a basis for the estimation of biotransformation of *P. capitatum in vivo*. 

## Supplementary Materials

Supplementary materials can be accessed at: http://www.mdpi.com/1420-3049/19/7/10291/s1.
