# Supplementary material for: Biotransformation and in Vitro Metabolic Profile of Bioactive Extracts from a Traditional Miao-Nationality Herbal Medicine, Polygonum capitatum
Source: Molecules. 2014 Jul 16;19(7):10291–308. doi: 10.3390/molecules190710291 (PMC6270779; doi:10.3390/molecules190710291)

Supplementary Information

Figure S1. HPLC chromatogram of metabolites in the incubation of extracts of *P. capitatum* in the intestinal bacteria *in vitro* (**a**) HPLC of metabolites of EtOAc fraction; (**b**): HPLC of metabolites of ethanol extracts; ——: incubation for 24 h; ----: negative control for 24 h; –––·: incubation for 0 h.

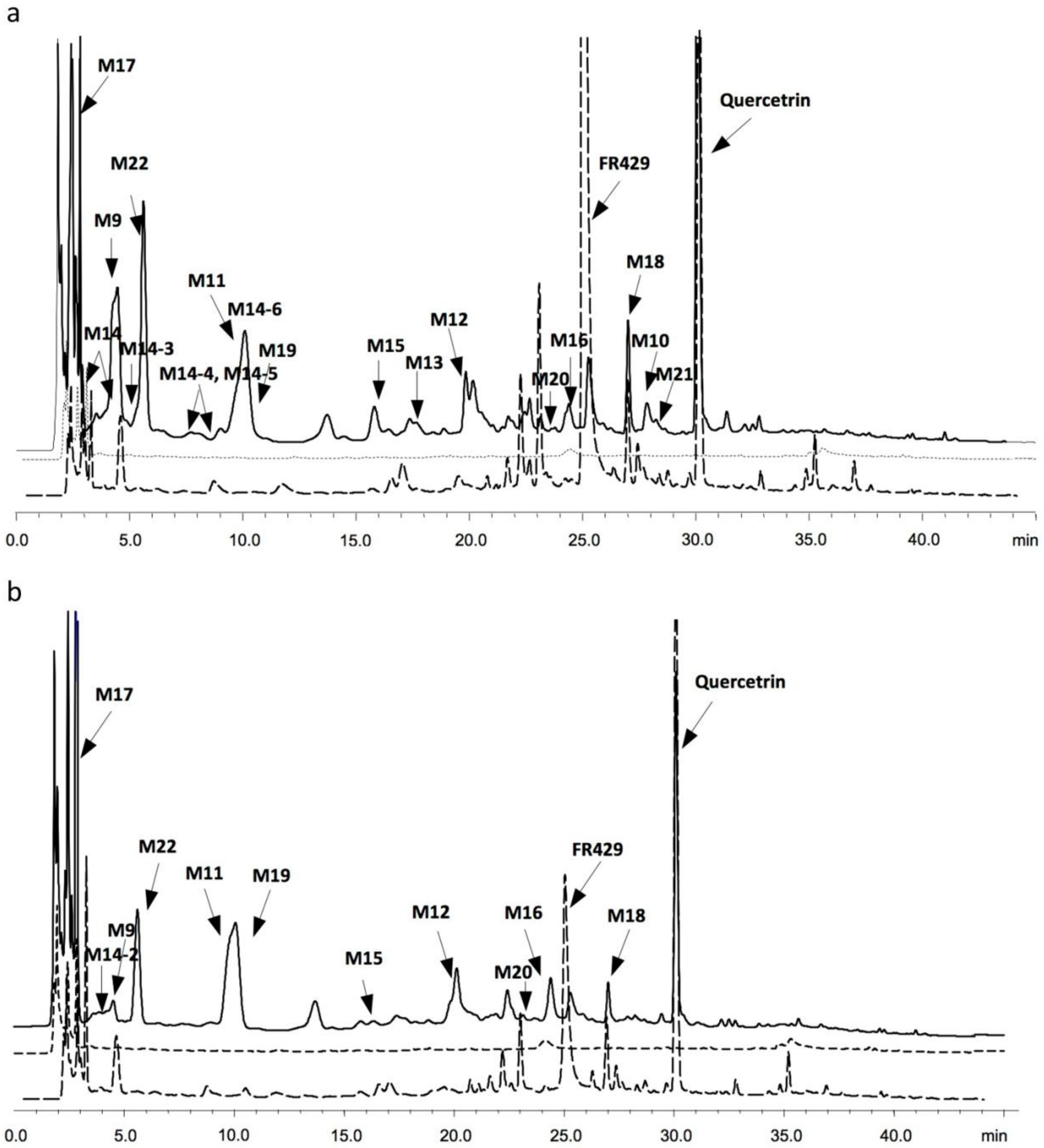

Supplement: Supplementary file 1 [file molecules-19-10291-s001.pdf]
